# A Case of Primary Intraosseous Adenoid Cystic Carcinoma of the Mandible

**DOI:** 10.1155/2023/2422086

**Published:** 2023-05-23

**Authors:** Erika Sasaki, Kenji Yamagata, Takayuki Hagiwara, Ryo Takasaki, Satoshi Fukuzawa, Fumihiko Uchida, Naomi Ishibashi-Kanno, Hiroki Bukawa

**Affiliations:** Department of Oral and Maxillofacial Surgery, Institute of Clinical Medicine, Faculty of Medicine, University of Tsukuba, Tsukuba, Ibaraki 305-8575, Japan

## Abstract

Primary intraosseous adenoid cystic carcinoma (PIACC) of the jaw is rare. To our knowledge, only 51 cases have been reported in the English literature. We present a rare case of PIACC arising in the mandible with multiple bone metastases and review the previous articles. A 70-year-old woman presented with paresthesia of the right chin and lower gingiva for 4 months. Radiography revealed an irregular radiolucent region on the right side of the ramus, infiltrating to the mandibular canal. Biopsy revealed a pathological diagnosis of adenoid cystic carcinoma. Multiple bone metastases were present in the sternum, scapula, and thighs. The treatment effect was progressive disease for chemotherapy; therefore, best supportive care was provided for 3 years.

## 1. Introduction

Intraoral adenoid cystic carcinoma (ACC) is a rare malignant epithelial neoplasm of salivary gland origin, characterized by slow evolution and indolent growth, multiple and delayed recurrences, late onset of distant metastasis, and poor prognosis [1]. In addition, primary intraosseous ACC (PIACC) of the jaw is rare. To our knowledge, only 51 cases have been previously reported from 1955 to 2022, with 47 cases from the review of Hu et al. (2017) and four recent cases reported in the English literature [2–6]. PIACC of the jaw has rarely been reported and is poorly understood. Here, we present a rare case of paresthesia of the inferior alveolar nerve with multiple bone metastases in a Japanese female patient and review previous reports in the literature.

## 2. Case Report

**Figure 1:**
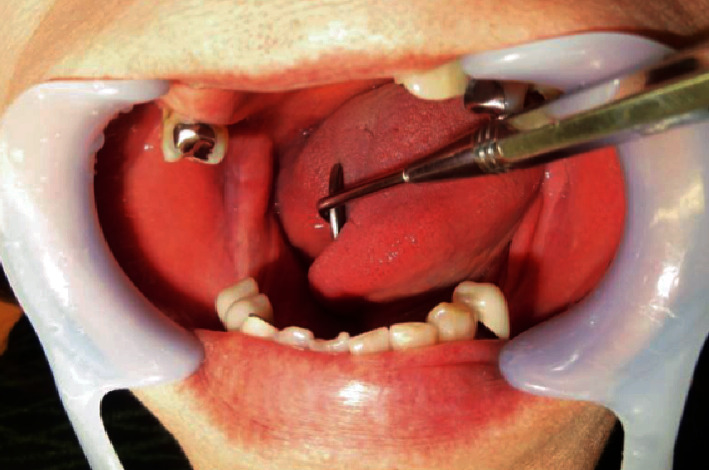
Oral findings. The right first and second molars were missed previously, and the gingival mucosa was clear and normal.

**Figure 2:**
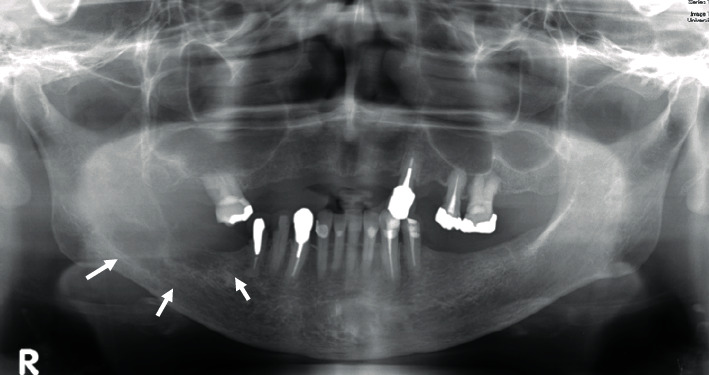
Panoramic radiograph. The panoramic radiograph revealed irregular radiolucent region on the right body to ramus infiltrating to the mandibular canal.

**Figure 3:**
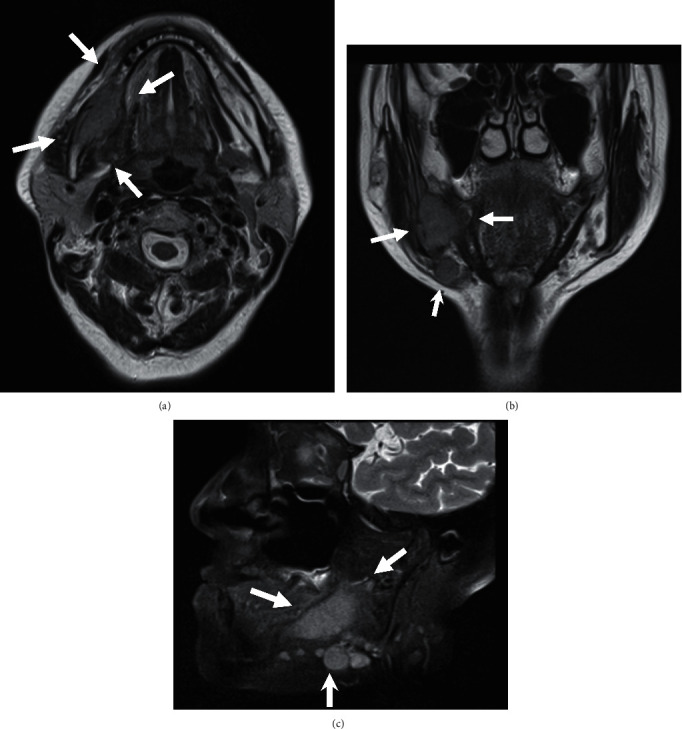
MR image. (a) Axial view. (b) Coronal view. (c) Sagittal view. T1-weighted MR reveals a 45 mm × 2 3 mm × 19 mm low-signal mass in the body of the mandible involving the mandibular canal. The submandibular LN is swollen.

**Figure 4:**
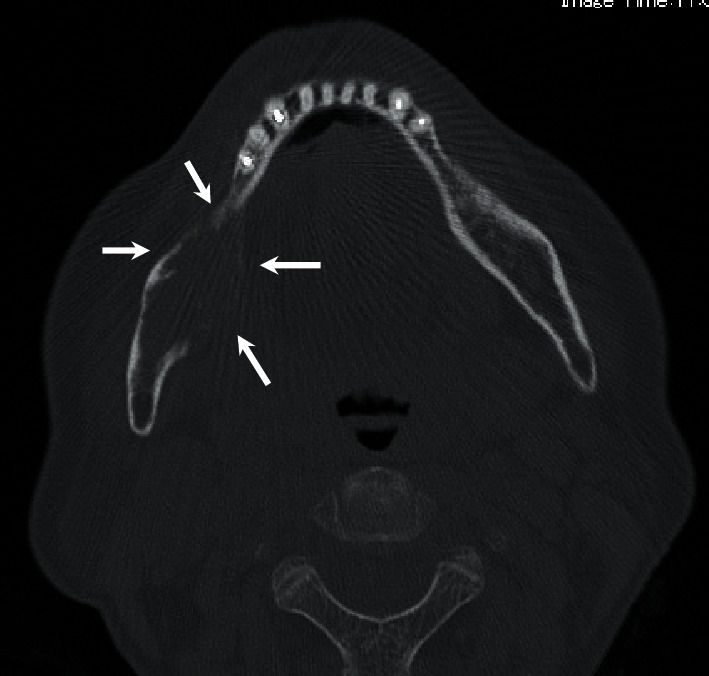
CT image. Axial view. CT depicts thin cortical bone and slightly partially destroyed lingual bone.

**Figure 5:**
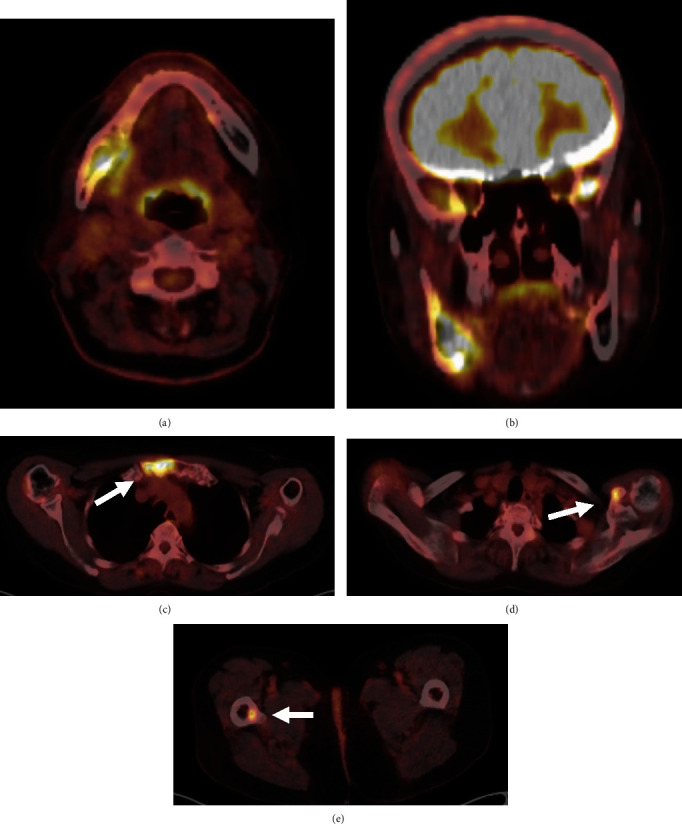
PET–CT imaging. PET imaging showed increased uptake in (a) and (b) right side of mandible, (c) left coracoid process of scapula, (d) sternum, and (e) right lesser trochanter of thigh bone.

**Figure 6:**
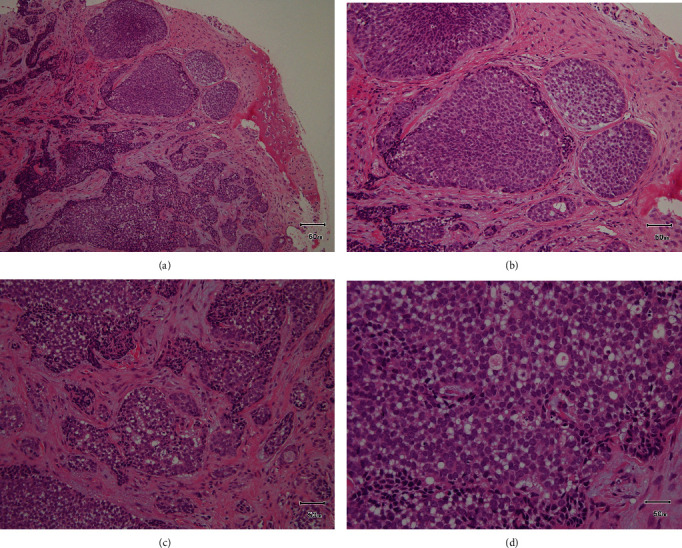
Pathological findings. HE staining: (a) low magnification view ×100, (b) solid alveolar like pattern ×200, (c) cribriform pattern ×200, and (d) high magnification view ×400. Pathologically, the tumor consists of a solid alveolar pattern with partially tubular and cribriform pattern formation. Interstitial hyalinization and myxomatous generation are observed.

**Figure 7:**
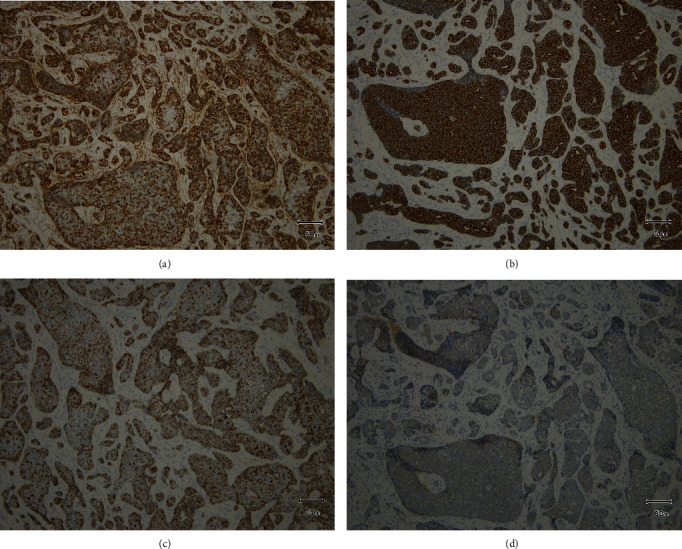
Immunohistochemistry results (low magnification view ×200). (a) *α*SMA. (b) Pankeratin. (c) EMA. (d) c-kit.

A 70-year-old Japanese woman was referred to the Department of Oral and Maxillofacial Surgery, University Hospital of Tsukuba, with a complaint of paresthesia in the inferior alveolar nerve region. Her medical history included hypertension, cerebral aneurysm, goiter, atrial fibrillation, and premature ventricular contraction. Paresthesia of the right chin and lower gingiva began 4 months prior, and she was examined with computed tomography (CT) at a general hospital. The CT revealed destruction of the right mandibular bone. Her general condition was good, and performance status was 0. Her face was symmetric, and trismus was absent. Paresthesia of the right inferior alveolar nerve was also observed. The right upper deep neck lymph nodes (LNs) and submandibular LN were elastic, hard, and swollen with sizes of 30 and 10 mm, respectively. The right first and second molars were previously missed, and the gingival mucosa was clear and normal (Figure 1). Panoramic radiography revealed an irregular radiolucent region extending from the right side of the mandibular body to the ramus infiltrating the mandibular canal (Figure 2). T1-weighted magnetic resonance (MR) revealed a 45 mm × 23 mm × 19 mm low-signal mass at the body of the mandible involving the mandibular canal. The submandibular LN was swollen (Figure 3). CT depicted a thin cortical bone and a slightly partially destroyed lingual bone (Figure 4). Chest radiography revealed no metastatic regions in the lungs, hilum, or mediastinal LNs. Positron emission tomography (PET)–CT revealed 20 mm ^18^F-fluorodeoxyglucose (FDG) accumulation in the right mandibular body and at the sternum, scapula, and thigh bone. Multiple bone metastases were suspected (Figure 5). Biopsies were performed under local anesthesia. The normal lower gingiva was incised, and a mucoperiosteal flap was elevated. Normal bone was defected at the alveolar crest, revealing a white elastic hard tumor. Pathologically, the tumor consisted of a solid alveolus like pattern with partially tubular and cribriform pattern formations. Hyalinization and myxomatous interstitial generations were also observed (Figure 6). Immunohistochemistry results were as follows: alpha smooth musle actin (*α*SMA) (+), pankeratin (+), epithelial membrane antigen (EMA) (+), c-kit (weak+), p40 (−), and p63 (−) (Figure 7). ACC was pathologically diagnosed. Based on these findings, the patient was clinically diagnosed with PIACC (T4aN2bM1).

**Figure 8:**
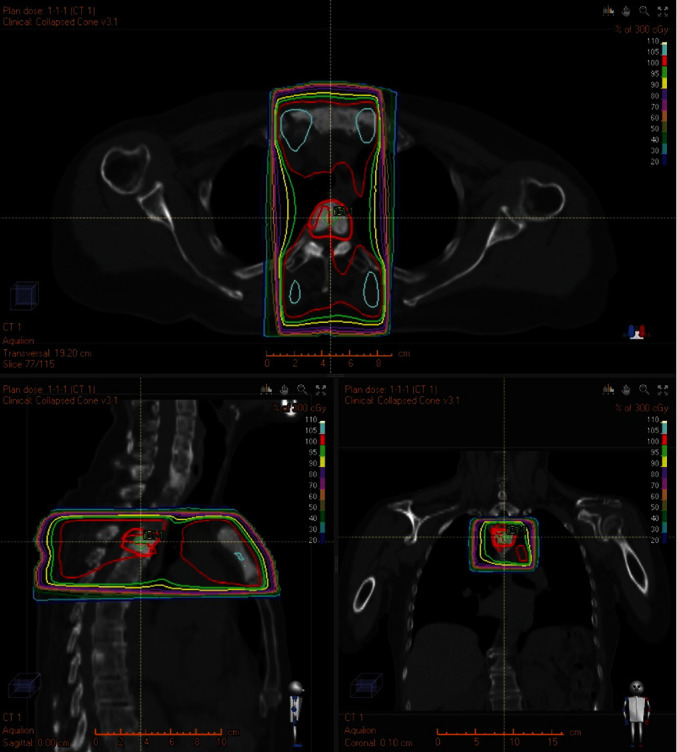
Field of palliative radiotherapy. Radiotherapy was performed for the third thoracic vertebrae epidural mass at 30 Gy/10 fractions.

A chemotherapy regimen of cisplatin, 5 fluorouracil (5FU), and cetuximab was administered in two courses because of the multiple bone metastases. Subsequently, three courses of cetuximab were administered. The treatment effect was progressive disease (PD), as revealed by PET–CT. Therefore, nivolumab was administered 12 times. Radiotherapy for the third thoracic vertebrae epidural mass was performed at 30 Gy/10 fractions (Figure 8). The patient was transferred to hospital for best supportive care (BSC) for 3 years from the first visit for PD.

## 3. Discussion

Our patient was the 52nd reported case of PIACC worldwide. ACC usually has a slow and insidiously malignant clinical course, characterized by microscopic foci of tumors that infiltrate the nerves, perineural space, and tissue planes, explaining the high incidence of recurrence. LN metastases are unusual, but hematogenous tumor spread to the lung is characteristic [7]. According to the nerve infiltrated, the PIACC arising in the mandible spreads along the inferior alveolar nerve. Paresthesia was described at an occurrence rate of 24.4% [2]. Therefore, paresthesia of the lower lip and mental region is a characteristic symptom of PIACC in the mandible, as in our case. Hu et al. reported that the PIACC of the mandible commonly occurs in the mandibular body and angle. The age of the patients peaked in the fourth to sixth decades. Pain and swelling are the most common symptoms. Almost all lesions showed radiolucent destruction of the bone, with an irregular margin [2]. In our case, the patient was in the sixth decade of life and the lesion extended from the mandibular body to the angle, which corresponded to previous reports.

The diagnosis of PIACC is difficult because of its rarity and lack of specific features compared with other intraosseous benign and malignant tumors. Strict diagnostic criteria for PIACC were reported in 1990, including radiographic evidence of osteolysis, intact cortical plates, absence of any primary lesion within the salivary glands or other tissues that resemble the architecture of salivary lesions, and histologic confirmation of ACC [7]. In the present study, CT of the axial view depicted thin partially destroyed lingual cortical plate because of enlargement of the tumor. The intraosseous mass was mainly presented in the body of the mandible in MR image. Therefore, it was strongly suggested that the mass was occurred from the intraosseous origin. The histogenesis of PIACC is unknown and controversial; the previous hypotheses presented: (1) presence of a Stafne bone defect or cavity in the lingual cortex of the mandible, occupied by salivary tissue; (2) the retromolar mucous gland, and submandibular and sublingual salivary glands may become embedded in the lingual cortex during embryological development of the jawbone, and intraosseous salivary gland tissue may be the origin of PIACC [2], and (3) neoplastic transformation of the odontogenic cyst epithelium has been suggested as the reason for PIACC [4]. Though the hypothesis of Stafne bone defect is anomaly to diagnostic criteria of PIACC because Stafne bone defect present outside of lingual cortex and no intraosseous lesion. Our case had no previous odontogenic cyst, and the lingual cortex was presented thin; moreover, a Stafne bone defect was not present. Therefore, the salivary glands during early embryologic development of the mandible were suggested to be the origin of the PIACC in this case.

Recently, Bajpai et al. reported the primary intraosseous basaloid squamous cell carcinoma [8]. It can be differentiated from PIACC by the absence of myoepithelial cells and positive expression of basaloid cells for p63. The immunohistochemical results presented *α*SMA (+) and p63 (−), and the different diagnosis was easy in our case.

PIACC is extremely rare and is often clinically misdiagnosed [9]. A high incidence of erroneous initial diagnoses was reported in 47 cases, including six cases of cystic lesions and ameloblastoma, four of periapical inflammation, one of odontogenic tumor, and one of periapical cemental dysplasia [2]. The patients were diagnosed with mandibular osteitis, odontogenic tumor (such as ameloblastoma), and cyst (such as odontogenic keratocyst) using panoramic radiograph. Hu et al. reported that ameloblastoma is a benign but locally aggressive epithelial neoplasm. It grows slowly and, in most cases, patients do not experience pain or paresthesia. Among the 47 cases of PIACC reported previously, six were initially diagnosed with ameloblastoma [2]. Therefore, in addition to radiography, the clinical symptoms of paresthesia led to the diagnosis of malignant tumor or osteomyelitis. In our case, MR image revealed a mass in the body of the mandible involving the mandibular canal and FDG–PET revealed multiple bone lesions with mandibular disease. Based on these examination results, a malignant diagnosis was relatively easy.

Hu et al. reported the pathological diagnosis of PIACC. Consistent with ACC arising in the salivary gland, three basic growth patterns of PIACC were identified: cribriform, tubular, and solid. Pathological information was available for 26 patients. The cribriform pattern was the most common in 12 patients, which was the best-recognized pattern and the prototypical pattern that typified the tumor. Tubular patterns were present in two patients. Three patients had cribriform and tubular features. The solid form was present in nine patients [2]. The histological subtype is one of the most important factors in prognosis, and the solid type develops distant metastasis most rapidly and has a poorer prognosis than those of the tubular and cribriform types [10]. Hu et al. reported the overall mean survival periods of without and with solid pattern were 57.3 and 23.6 months, respectively. The determinant 5-year survival rate without and with a solid pattern was 83.3% and was less than 5 years [2]. In our patient, the tumor consisted of a solid alveolar pattern with partially tubular and cribriform pattern formations. Multiple bone metastases were observed during the first visit because of a solid pathological pattern. The survival of our patient was more than 3 years on BSC and longer than that in the solid pattern report. This may be because our patient presented tubular and cribriform pattern formations.

Previous reports have recommended radical surgery combined with postsurgical radiotherapy for the best treatment [2, 11]. ACC has a strong propensity to infiltrate adjacent tissues and nerves with a high incidence of recurrence; therefore, wide and clear surgical margins were ensured. Moreover, hematogenous tumor spread to the lung is quite characteristic [7], and radical surgery is difficult in some cases. Our patient had multiple bone metastases, and surgery was not indicated. Although conventional chemotherapy and immune checkpoint inhibitors (ICIs) were administered, cisplatin, 5FU, cetuximab, and secondary nivolumab were administered. The treatment effect was PD to both chemotherapy regimens and change to BSC. Laurie et al. reported that chemotherapy is generally reserved for palliative treatment of symptomatic locally recurrent or metastatic disease that is not amenable to further surgery or radiation [12]. Lorini et al. reported that owing to disappointing results with chemotherapy, new approaches based on biomolecular research are under study. Prospective trials of chemotherapy for advanced ACC are limited, and the optimal regimen is unclear. Ongoing clinical trials have evaluated treatments targeting MYB and NOTCH1 alterations, immunotherapy, or a combination of targeted treatments and ICIs [13].

## Data Availability

Data supporting this research article are available from the corresponding author or first author on reasonable request.
